# Supplementary material for: Structural insights into AQP3 channel closure upon pH and redox changes reveal an autoregulatory molecular mechanism
Source: Nat Commun. 2025 Dec 22;16:10997. doi: 10.1038/s41467-025-67144-2 (PMC12722722; doi:10.1038/s41467-025-67144-2)
Supplement: Supplementary file 1 — Supplementary Information [file 41467_2025_67144_MOESM1_ESM.docx]

**Structural insights into AQP3 channel closure upon pH and redox changes reveal an autoregulatory molecular mechanism**

Peng Huang^1,‡^, Raminta Venskutonytė^1,2,‡^, Carter J. Wilson^3,‡^, Sara Bsharat^4^, Rashmi B. Prasad^4^, Pontus Gourdon^1,5^, Isabella Artner^4^, Bert L. de Groot^3^, Karin Lindkvist-Petersson^1,2,*^

1. Department of Experimental Medical Science, Lund University, Lund, Sweden.
2. LINXS - Institute of Advanced Neutron and X-ray Science, Lund, Sweden.
3. Computational Biomolecular Dynamics Group, Max Planck Institute for Multidisciplinary Sciences, 37077 Gottingen, Germany
4. Lund University Diabetes Centre, Clinical Research Center, Malmo, Sweden.
5. Department of Biomedical Sciences, Copenhagen University, Maersk Tower 7-9, Nørre Allé 14, DK-2200, Copenhagen N, Denmark

^*^To whom correspondence may be addressed: Prof. Karin Lindkvist-Petersson, Department of Experimental Medical Science, Lund University, BMC C13, 221 84 Lund, Sweden, +46 46 2228041, E-mail: [karin.lindkvist@med.lu.se](mailto:karin.lindkvist@med.lu.se)

^‡^contributed equally


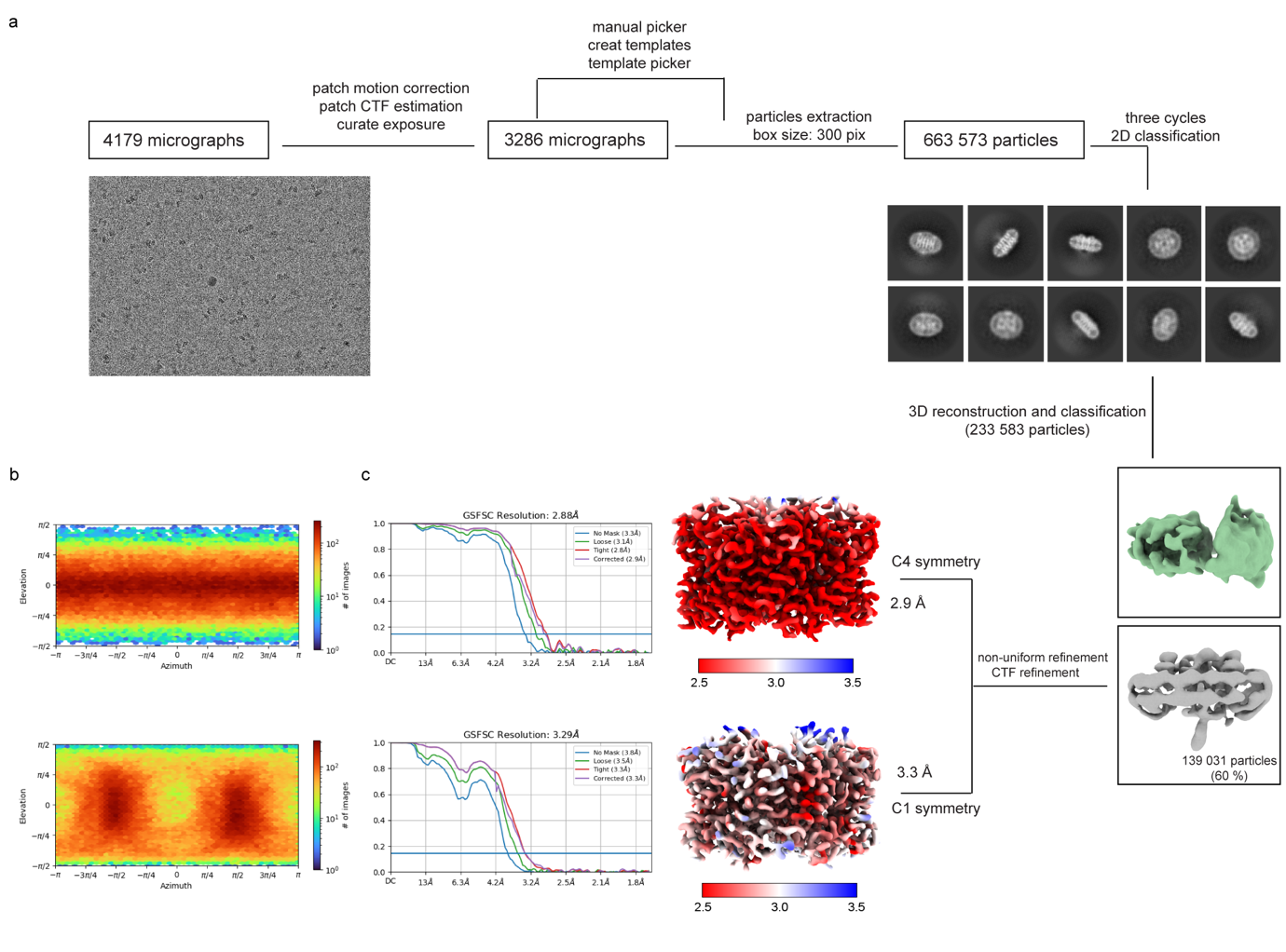


**Figure S1.** **Cryo-EM data processing summary in CryoSPARC for the AQP3 at pH 8.0.** 3D reconstruction resulted in two models, and one was refined to high quality maps, both with C1 and with C4 symmetry applied.


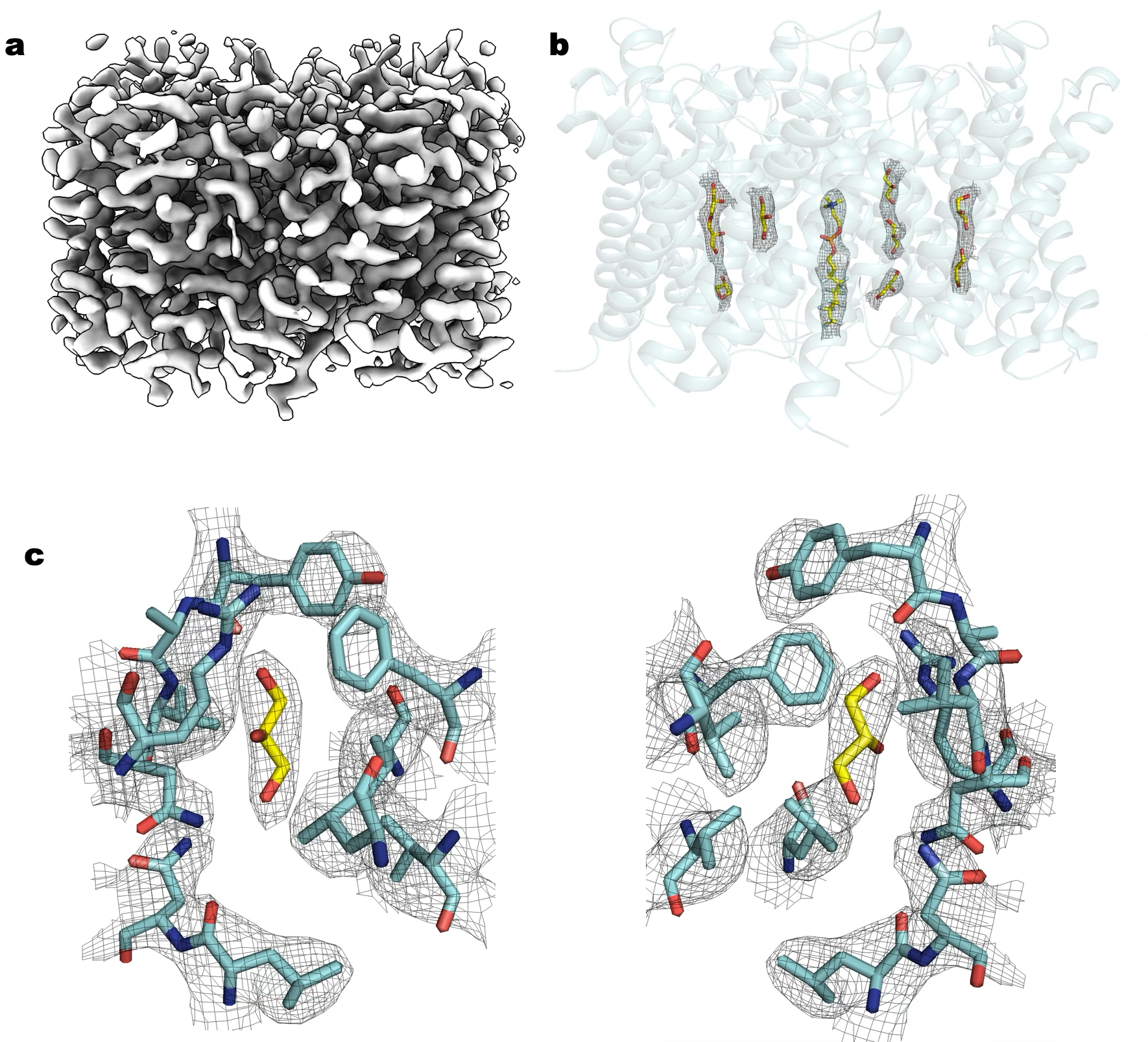


**Figure S2. Cryo-EM densities of ligands at pH 8.0**. (a) Side view of the cryo-EM map. (b) AQP3 tetramer side view with glycerol molecules modelled within the individual channels of each monomer and an FF8 molecule modelled in the central pore. Ligand cryo-EM density is shown in mesh. (c) zoom in of the glycerol and the surrounding residues (chain A) at two different angles with cryo-EM density shown in mesh.


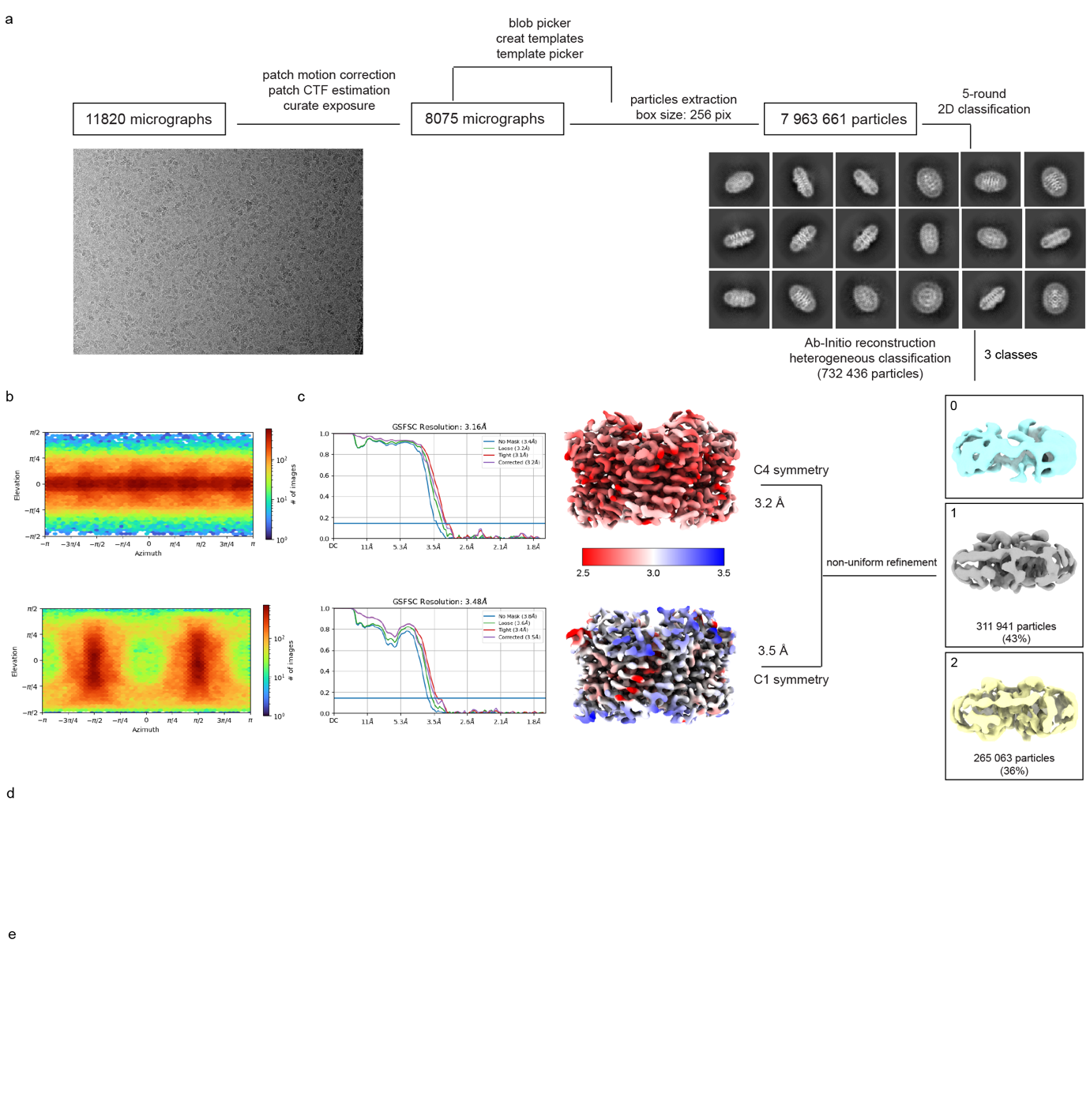


**Figure S3. Cryo-EM data processing summary in CryoSPARC for the AQP3 at pH 5.5.** 3D reconstruction resulted in three models, and one was refined to high quality maps, both with C1 and with C4 symmetry applied.


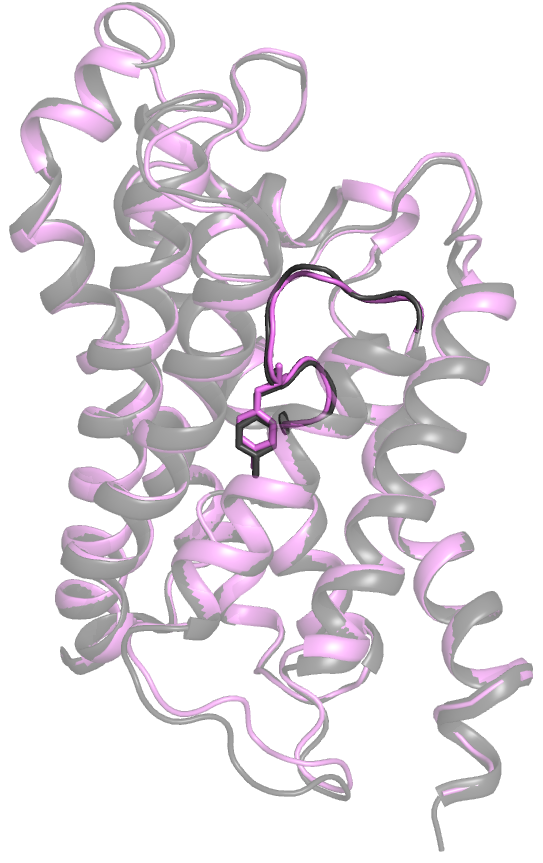


**Figure S4. Comparison of the closed human AQP3 cryo-EM structure and rat AQP3 cryo-EM structure.** Cartoon representation of the aligned human AQP3 structure at pH 5.5 (pink) and rat AQP3 structure (gray, PDB ID:8Y8O). Loop E and Tyr212 are shown in deeper colours.


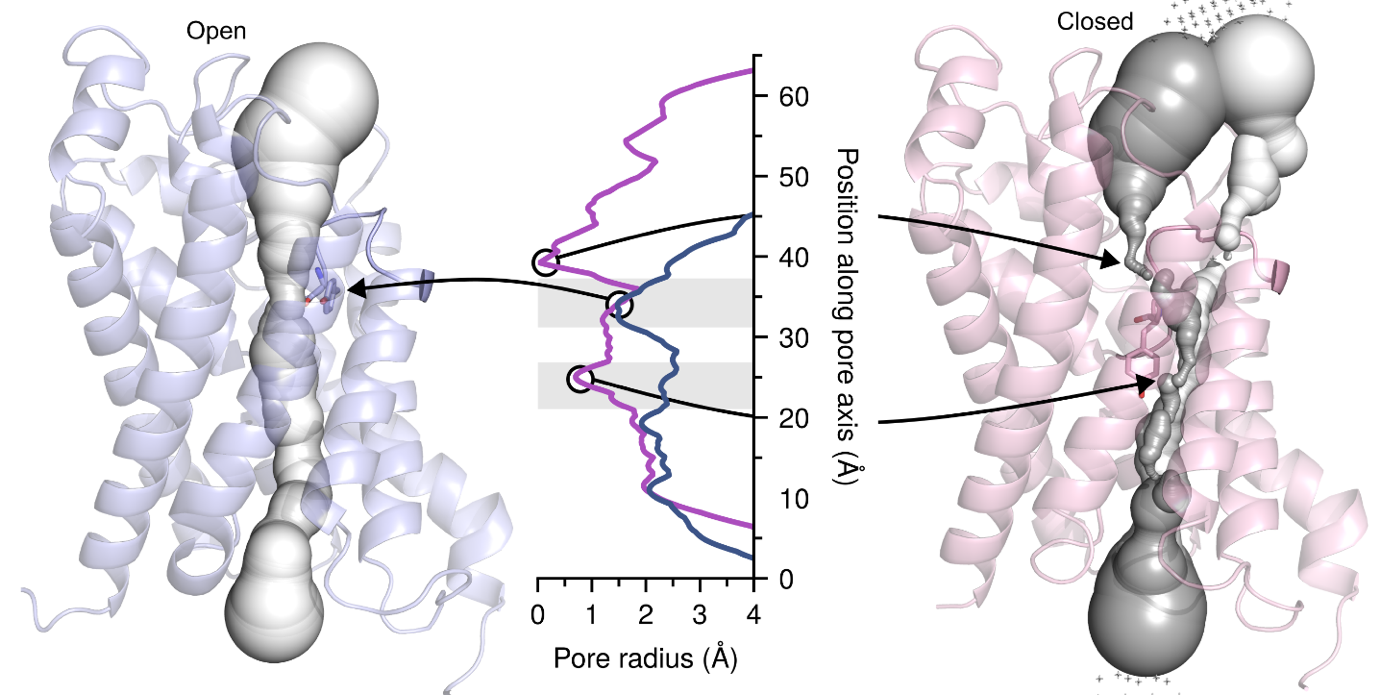


**Figure S5. HOLE analysis**. Depiction of the open in cartoon representation (pH 8, blue) and closed (pH 5.5, magenta) channel alongside HOLE pore analysis. For the closed channel HOLE was run in both directions yielding similar blocks. Constriction regions for the closed channel are indicated. Removal of the E loop creates a valid water path and the pore radius beyond the constriction is similar to the open channel. Tyr212 is shown in stick representation.


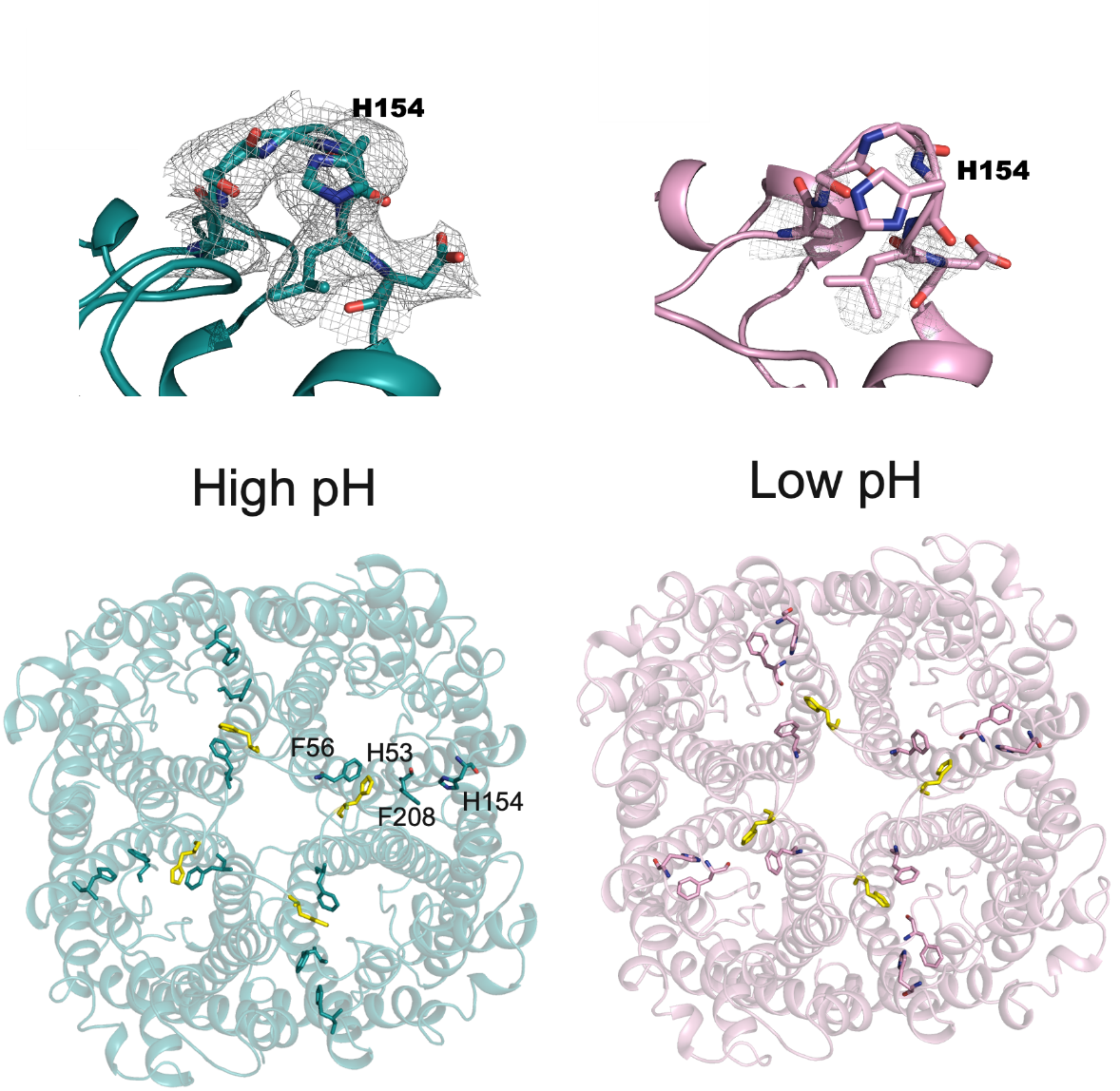


**a**

**b**


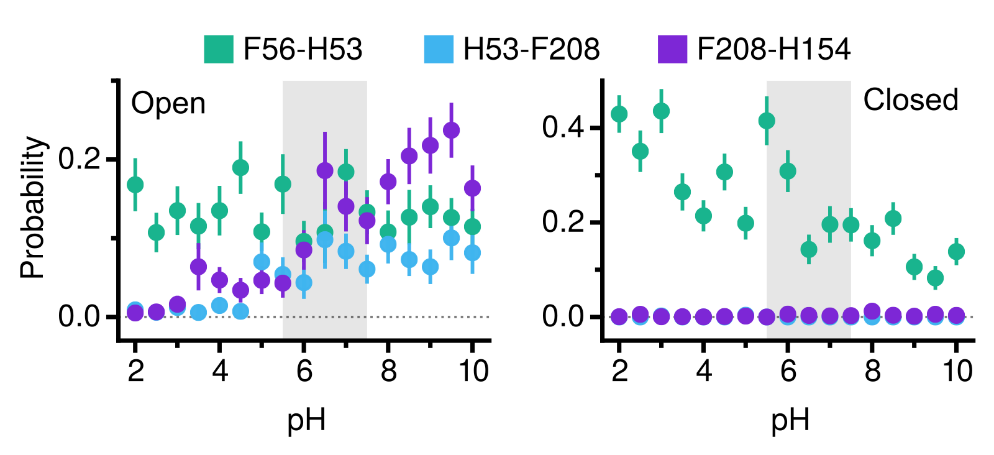


**Figure S6. The tetrad**. (a) Upper panel: Loop C with cryo-EM density shown for the pH 8 (cyan) and the pH 5.5 (pink) structures. Lower panel: View from the extracellular side with residues of the HFHF tetrad shown in sticks for the pH 8 (cyan) and pH 5.5 (pink) structures. (b) pH-dependent interaction propensities between various tetrad pairs for the open (pH 8) and closed (pH 5.5) states across the CpHMD simulations.


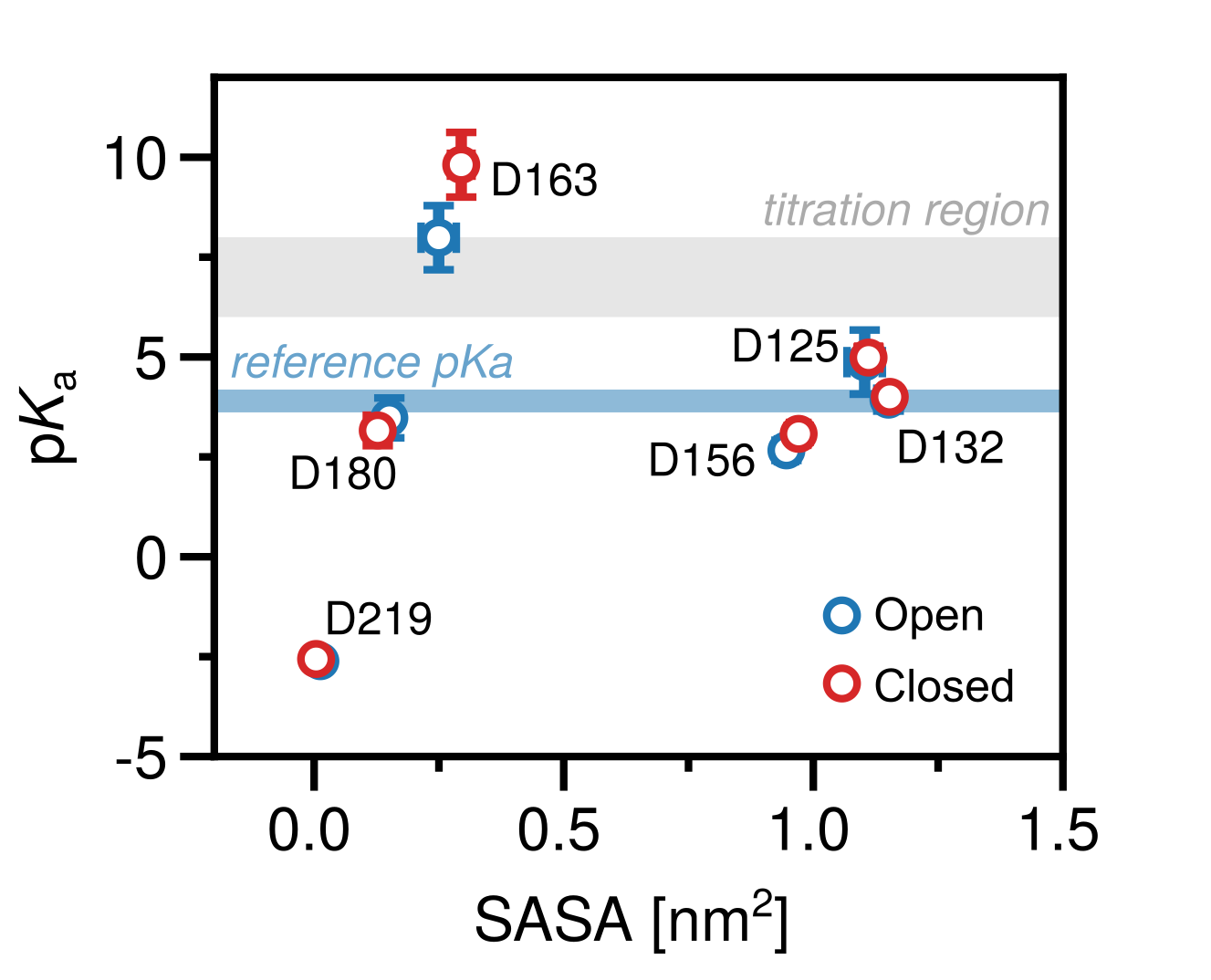


**Figure S7 Solvent-accessible surface area (SASA) for titratable residues near loop E.** The calculated pKa values for titratable residues near loop E in the pH 8.0 (open) structure shown by blue circles, and in the low pH structure at 5.5 (closed) in red circles.


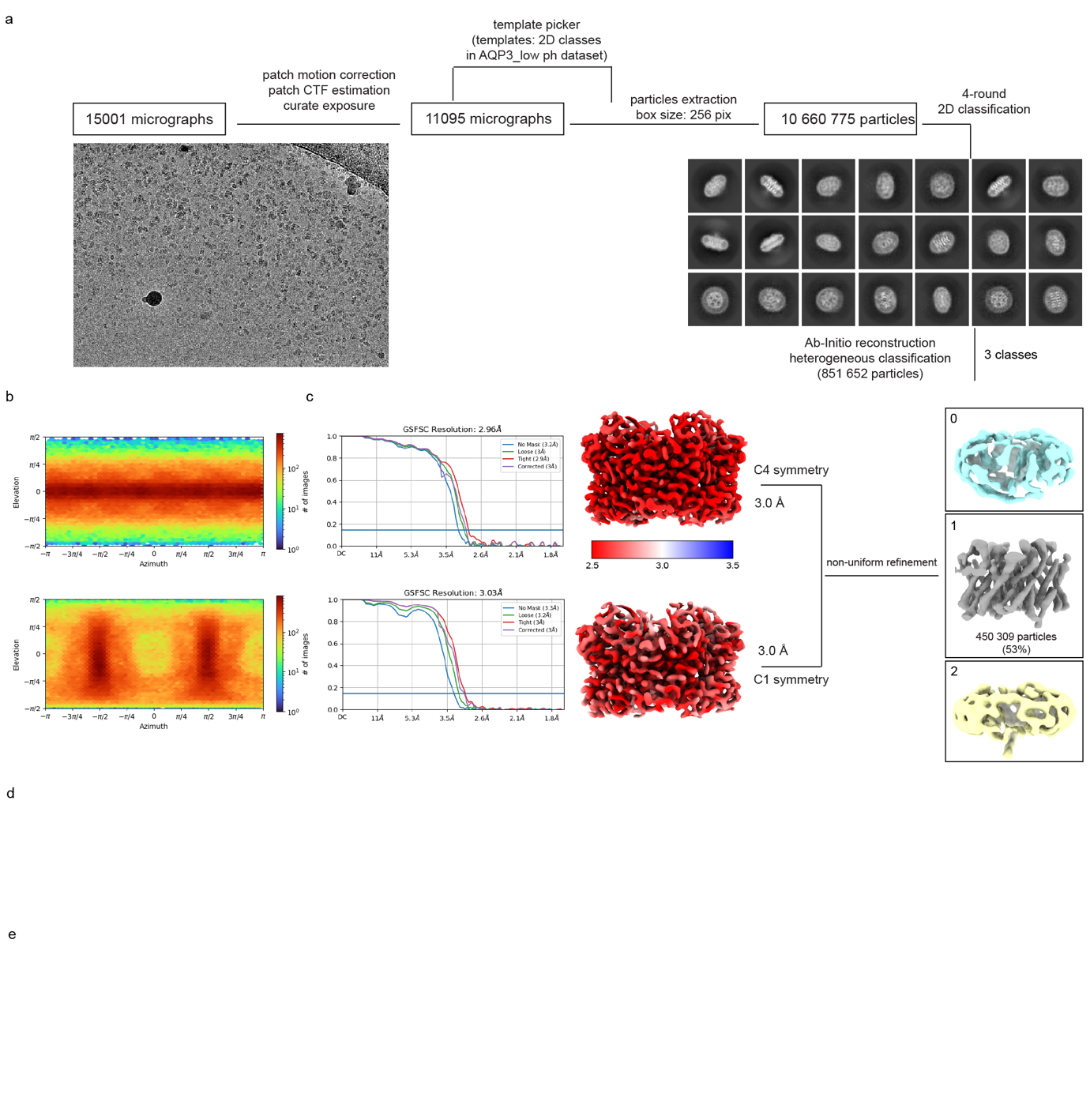


**Figure S8. Cryo-EM data processing summary in CryoSPARC for the AQP3 in the presence of H_2_O_2_.** 3D reconstruction resulted in three models, and one was refined to high quality maps, both with C1 and with C4 symmetry applied.


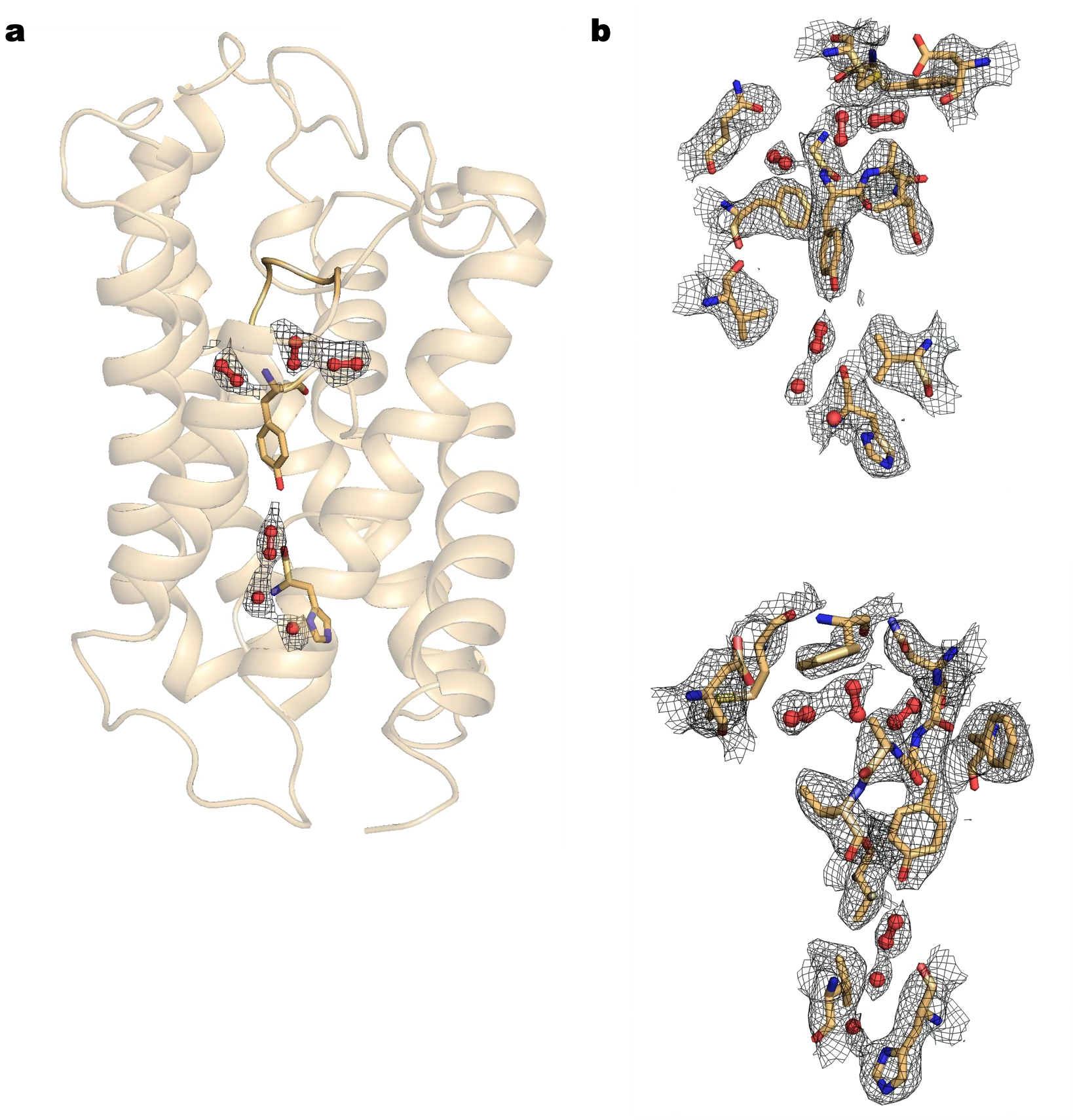


**Figure S9. H_2_O_2_ cryo-EM density in AQP3**. (a) AQP3 shown as cartoon (beige), Tyr212, His81, hydrogen peroxide and water (red) shown as sticks. (b) Zoom-in of the H_2_O_2_, water molecules and the surrounding residues at two different angles with cryo-EM density shown as mesh.


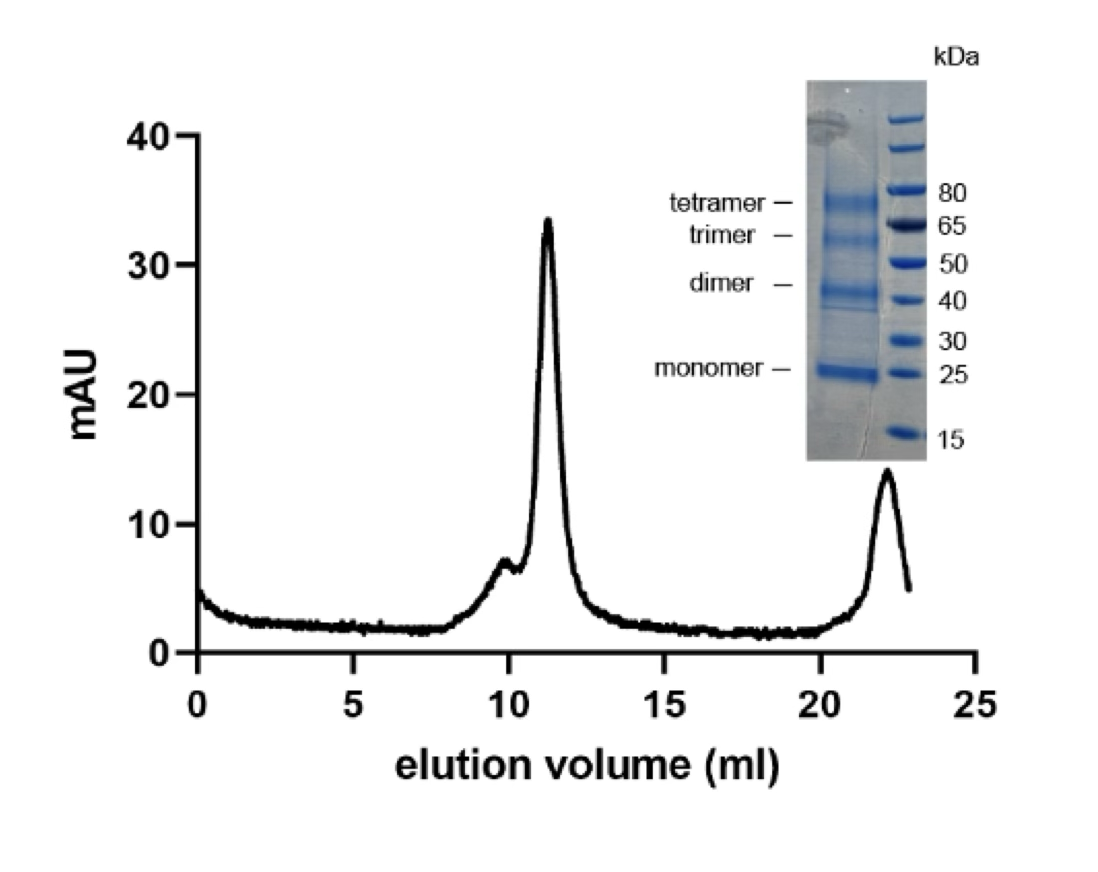


**Figure S10. Size exclusion chromatography profile and SDS-PAGE of AQP3.** AQP3 reconstituted from DDM into nanodiscs. The peak fractions were used for cryo-EM grid preparation.


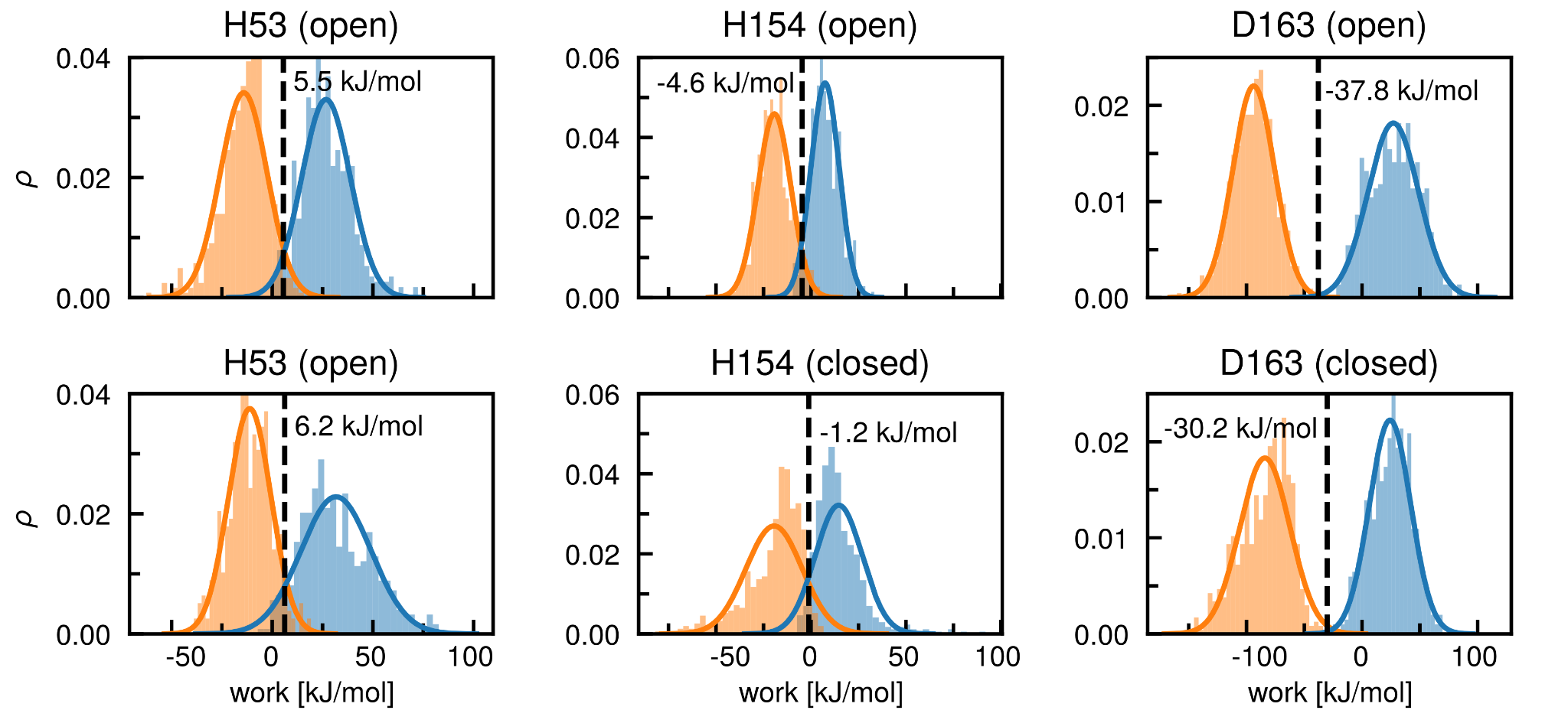


H53 (closed)

**Figure 11 Convergence analyses**. The converged overlap of the work distributions for the three probed pKa values in the two states (open and closed).

Table S1. Cryo-EM data and processing parameters and refinement statistics.

|  | AQP3-high pH | AQP3-low pH | AQP3-H_2_O_2_ |
| --- | --- | --- | --- |
| **Data collection and processing** | | | |
| Microscopy | FEI Titan Krios FEI Titan Krios FEI Titan Krios | | |
| Voltage (kV) | 300 300 300 | | |
| Total exposure dose (e¯/ Å^2^) | 39.866 | 52.825 | 52.825 |
| Frame | 40 | 40 | 40 |
| Pixel size (Å) | 0.85 | 0.83 | 0.83 |
| Data process software | cryoSPARC | cryoSPARC | cryoSPARC |
| Symmetry imposed | C1 | C4 | C4 |
| Map resolution (Å) | 3.3 | 3.2 | 3.0 |
| FSC threshold | 0.143 | 0.143 | 0.143 |
| **Refinement** |  |  |  |
| Map sharpening B factor (Å) | 120 | 174.5 | 149.3 |
| Model composition |  |  |  |
| Nonhydrogen atoms | 7741 | 7576 | 7568 |
| Protein residues | 1013 | 1004 | 1000 |
| Water | 2 | 4 | 12 |
| Glycerol/FF8/PEO | 8/1/- | - | -/-/16 |
| Mean B-factors (Å^2^) |  |  |  |
| Protein | 51.20 | 64.54 | 39.72 |
| Ligands | 58.29 | - | 37.49 |
| Water | 36.46 | 32.6 | 28.99 |
| Bonds (RMSD) |  |  |  |
| Length (Å) | 0.002 | 0.003 | 0.002 |
| Angles (°) | 0.499 | 0.495 | 0.452 |
| **Validation** |  |  |  |
| MolProbity score | 1.84 | 1.56 | 1.59 |
| Clashscore | 5.30 | 3.5 | 5.24 |
| Rotamer outliers | 1.54 | 1.55 | 0.52 |
| Ramachandran plot |  |  |  |
| Favored (%) | 93.83 | 95.98 | 95.56 |
| Allowed (%) | 6.17 | 4.02 | 4.44 |
| Disallowed (%) | 0 | 0 | 0 |

Table S2. Real – space correlation coefficient (CC) for modelled ligands and water molecules as calculated by Phenix Real-space refinement.

| **AQP3 pH 8.0** | | **AQP3 pH 5.5** | | **AQP3 – H_2_O_2_** | |
| --- | --- | --- | --- | --- | --- |
| Overall | 0.85 | Overall | 0.82 | Overall | 0.87 |
| A 304 GOL | 0.80 | A 301 HOH | 0.58 | A 301 PEO | 0.84 |
| A 306 LIG | 0.73 |  |  | A 401 PEO | 0.78 |
| D 301 GOL | 0.78 |  |  | A 501 PEO | 0.65 |
| D 302 GOL | 0.66 |  |  | A 601 PEO | 0.76 |
| D 303 GOL | 0.64 |  |  | E 1 HOH | 0.76 |
| C 401 GOL | 0.81 |  |  | E 2 HOH | 0.76 |
| C 401 GOL | 0.77 |  |  | E 3 HOH | 0.74 |
| B 301 GOL | 0.75 |  |  |  |  |
| B 302 GOL | 0.81 |  |  |  |  |
| E 16 HOH | 0.66 |  |  |  |  |
| E 17 HOH | 0.62 |  |  |  |  |

Table S3. System composition for MD simulations

|  | Box size (nm) | H_2_O molecules | Total atoms | Ions |
| --- | --- | --- | --- | --- |
| AQP3 tetramer with 172 POPC lipids | 9.9 x 9.9 x 9.5 | 17313 | 90675 | K^+^ : 56  Cl^-^ : 52 |
