# Supplementary material for: Structural insights into AQP3 channel closure upon pH and redox changes reveal an autoregulatory molecular mechanism
Source: Nat Commun. 2025 Dec 22;16:10997. doi: 10.1038/s41467-025-67144-2 (PMC12722722; doi:10.1038/s41467-025-67144-2)
Supplement: Supplementary file 2 — Reporting Summary [file 41467_2025_67144_MOESM2_ESM.pdf]

nature portfolio

Corresponding author(s): Karin Lindkvist-Petersson

Last updated by author(s): Aug 19, 2025

Reporting Summary

Nature Portfolio wishes to improve the reproducibility of the work that we publish. This form provides structure for consistency and transparency in reporting. For further information on Nature Portfolio policies, see our Editorial Policies and the Editorial Policy Checklist.

Statistics

For all statistical analyses, confirm that the following items are present in the figure legend, table legend, main text, or Methods section.

n/a	Confirmed	
☐	☒	The exact sample size (*n*) for each experimental group/condition, given as a discrete number and unit of measurement
☐	☒	A statement on whether measurements were taken from distinct samples or whether the same sample was measured repeatedly
☐	☒	The statistical test(s) used AND whether they are one- or two-sided<br>*Only common tests should be described solely by name; describe more complex techniques in the Methods section.*
☐	☒	A description of all covariates tested
☐	☒	A description of any assumptions or corrections, such as tests of normality and adjustment for multiple comparisons
☐	☒	A full description of the statistical parameters including central tendency (e.g. means) or other basic estimates (e.g. regression coefficient) AND variation (e.g. standard deviation) or associated estimates of uncertainty (e.g. confidence intervals)
☐	☒	For null hypothesis testing, the test statistic (e.g. *F*, *t*, *r*) with confidence intervals, effect sizes, degrees of freedom and *P* value noted<br>*Give P values as exact values whenever suitable.*
☒	☐	For Bayesian analysis, information on the choice of priors and Markov chain Monte Carlo settings
☒	☐	For hierarchical and complex designs, identification of the appropriate level for tests and full reporting of outcomes
☒	☐	Estimates of effect sizes (e.g. Cohen's *d*, Pearson's *r*), indicating how they were calculated

Our web collection on statistics for biologists contains articles on many of the points above.

Software and code

Policy information about availability of computer code

Data collection	Single particle cryo-EM data were collected on Titan Krios electron microscopes with a K3 detector.
Data analysis	Cryo-EM data were processed with cryosparc (version 2.15.0, https://cryosparc.com). Model building was performed using USCF chimera 1.14, Wincoot 0.9.2, Coot 0.9.8.93 , and phenix 1.21.2_5419) Figures were generated using pymol (version 3.1.3) and ChimeraX (version 1.6.1).

For manuscripts utilizing custom algorithms or software that are central to the research but not yet described in published literature, software must be made available to editors and reviewers. We strongly encourage code deposition in a community repository (e.g. GitHub). See the Nature Portfolio guidelines for submitting code & software for further information.

Data

Policy information about availability of data

All manuscripts must include a data availability statement. This statement should provide the following information, where applicable:

- Accession codes, unique identifiers, or web links for publicly available datasets
- A description of any restrictions on data availability
- For clinical datasets or third party data, please ensure that the statement adheres to our policy

The atomic coordinates and the corresponding cryo-EM maps have been deposited in the Protein Data Bank (PDB) and in the Electron Microscopy Data Bank (EMDB) under accession codes 9QSX [https://doi.org/10.2210/pdb9qsx/pdb] and EMD-53343 [https://www.ebi.ac.uk/pdbe/entry/emdb/EMD-53343] for AQP3 at pH 8.0; 9QSY [https://doi.org/10.2210/pdb9qsy/pdb] and EMD-53344 [https://www.ebi.ac.uk/pdbe/entry/emdb/EMD-53344] for AQP3 at pH 5.5; and 9QSZ

April 2023

[https://doi.org/10.2210/pdb9qsz/pdb] and EMD-53345 [https://www.ebi.ac.uk/pdbe/entry/emdb/EMD-53345] for AQP3-H2O2. Simulation setup files are available at: https://github.com/deGrootLab/aquaporin_3_2025. Source data are provided with this article.

Research involving human participants, their data, or biological material

Policy information about studies with human participants or human data. See also policy information about sex, gender (identity/presentation), and sexual orientation and race, ethnicity and racism.

Reporting on sex and gender	This has been reported in: Islet Gene View-a tool to facilitate islet research. Asplund O, Storm P, Chandra V, Hatem G, Ottosson-Laakso E, Mansour-Aly D, Krus U, Ibrahim H, Ahlqvist E, Tuomi T, Renström E, Korsgren O, Wierup N, Ibberson M, Solimena M, Marchetti P, Wollheim C, Artner I, Mulder H, Hansson O, Otonkoski T, Groop L, Prasad RB.Life Sci Alliance. 2022 Aug 10;5(12):e202201376. doi: 10.26508/lsa.202201376.
Reporting on race, ethnicity, or other socially relevant groupings	*Please specify the socially constructed or socially relevant categorization variable(s) used in your manuscript and explain why they were used. Please note that such variables should not be used as proxies for other socially constructed/relevant variables (for example, race or ethnicity should not be used as a proxy for socioeconomic status).* *Provide clear definitions of the relevant terms used, how they were provided (by the participants/respondents, the researchers, or third parties), and the method(s) used to classify people into the different categories (e.g. self-report, census or administrative data, social media data, etc.)* *Please provide details about how you controlled for confounding variables in your analyses.*
Population characteristics	The characteristics are previously published: Islet Gene View-a tool to facilitate islet research. Asplund O, Storm P, Chandra V, Hatem G, Ottosson-Laakso E, Mansour-Aly D, Krus U, Ibrahim H, Ahlqvist E, Tuomi T, Renström E, Korsgren O, Wierup N, Ibberson M, Solimena M, Marchetti P, Wollheim C, Artner I, Mulder H, Hansson O, Otonkoski T, Groop L, Prasad RB.Life Sci Alliance. 2022 Aug 10;5(12):e202201376. doi: 10.26508/lsa.202201376.
Recruitment	Cadaver
Ethics oversight	Human pancreas was obtained from the Human Tissue Laboratory, which is funded by the Excellence Of Diabetes Research in Sweden (EXODIAB) network (www.exodiab.se/home) in collaboration with The Nordic Network for Clinical Islet Transplantation Program (www.nordicislets.org). Informed consent was obtained from pancreatic donors or their relatives and all procedures were approved by the Swedish Ethical Review Authority (Permit number 2011263).

Note that full information on the approval of the study protocol must also be provided in the manuscript.

Field-specific reporting

Please select the one below that is the best fit for your research. If you are not sure, read the appropriate sections before making your selection.

[x] Life sciences [ ] Behavioural & social sciences [ ] Ecological, evolutionary & environmental sciences

For a reference copy of the document with all sections, see nature.com/documents/nr-reporting-summary-flat.pdf

Life sciences study design

All studies must disclose on these points even when the disclosure is negative.

Sample size	Complete cryo-EM statistics are provided in the Methods section, Extended data Figs. 1,3,8. Each structure is determined from particles from one selected grid. All samples available for the human cadavers were included in the studies. The number of samples for analysis of expression in humans was dictated by the availability of human donor tissue. AQP3 protein expression levels in human islets was normalized towards background expression in exocrine tissue to ensure reproducibility.
Data exclusions	No data was excluded.
Replication	AQP3 proteins were produced and purified multiple times in different conditions successfully. Multiple cryo-EM grids were prepared and frozen for AQP3, data was collected with one selected grid for each sample, and all data are included in the data analysis.<br><br>Tissue was collected from non-diabetic and diabetic donors. Non-diabetic donors were defined as individuals lacking the diagnosis of diabetes and with an Hba1c <42mmol/mol (6.0%). Diabetic donors had been clinically diagnosed with diabetes prior to hospital admission. 188 human donor islets were collected for expression analysis. We analyzed 3-4 non diabetic and diabetic pancreas for immunohistochemistry.
Randomization	Extracted particles were randomly assigned to calculate gold-standard FSC.
Blinding	No blinding was applied as no group allocation was used

Reporting for specific materials, systems and methods

We require information from authors about some types of materials, experimental systems and methods used in many studies. Here, indicate whether each material, system or method listed is relevant to your study. If you are not sure if a list item applies to your research, read the appropriate section before selecting a response.

Materials & experimental systems

n/a	Involved in the study
☐	☒ Antibodies
☒	☐ Eukaryotic cell lines
☒	☐ Palaeontology and archaeology
☒	☐ Animals and other organisms
☒	☐ Clinical data
☒	☐ Dual use research of concern
☒	☐ Plants

Methods

n/a	Involved in the study
☒	☐ ChIP-seq
☒	☐ Flow cytometry
☒	☐ MRI-based neuroimaging

Antibodies

Antibodies used	The primary antibodies with the following dilutions were used: insulin (1:1,000. Dako, Cat#506442), AQP3 (1:100. Abcam, Cat#ab125219) and glucagon (1:1000. Abcam, Cat#ab10988). Secondary antibodies used were: Cy2-, Cy3-, and Cy5- conjugated α-guinea pig, α-mouse, and α-rabbit (1:500. Jackson ImmunoResearch, Cat#s: 711-165-152, 712-176-153, 706-225-148, 715-225-150, 715-175-150). Nuclear staining was performed using DAPI (1:6000. Invitrogen).
Validation	All antibodies have been validated by respective manufacturers at least by Western blot. The secondary antibodies have been routinely used in our lab and produce reliable results with multiple primary antibodies. All antibodies have also been reported in previous studies with different species (including human and mouse) and we have confirmed expression patterns and subcellular localization.

Plants

Seed stocks	*Report on the source of all seed stocks or other plant material used. If applicable, state the seed stock centre and catalogue number. If plant specimens were collected from the field, describe the collection location, date and sampling procedures.*
Novel plant genotypes	*Describe the methods by which all novel plant genotypes were produced. This includes those generated by transgenic approaches, gene editing, chemical/radiation-based mutagenesis and hybridization. For transgenic lines, describe the transformation method, the number of independent lines analyzed and the generation upon which experiments were performed. For gene-edited lines, describe the editor used, the endogenous sequence targeted for editing, the targeting guide RNA sequence (if applicable) and how the editor was applied.*
Authentication	*Describe any authentication procedures for each seed stock used or novel genotype generated. Describe any experiments used to assess the effect of a mutation and, where applicable, how potential secondary effects (e.g. second site T-DNA insertions, mosiacism, off-target gene editing) were examined.*
